# Synthesis and Biological Properties of Fungal Glucosylceramide

**DOI:** 10.1371/journal.ppat.1003832

**Published:** 2014-01-09

**Authors:** Maurizio Del Poeta, Leonardo Nimrichter, Marcio L. Rodrigues, Chiara Luberto

**Affiliations:** 1 Department of Molecular Genetics and Microbiology, Stony Brook University, Stony Brook, New York, United States of America; 2 Instituto de Microbiologia Professor Paulo de Góes, Universidade Federal do Rio de Janeiro, Rio de Janeiro, Brazil; 3 Fundação Oswaldo Cruz – Fiocruz, Centro de Desenvolvimento Tecnológico em Saúde (CDTS), Rio de Janeiro, Brazil; 4 Department of Physiology and Biophysics, Stony Brook University, Stony Brook, New York, United States of America; Duke University Medical Center, United States of America

## Introduction

Sphingolipids have recently emerged as key regulators of pathogenicity in a variety of fungi. Glucosylceramide is a sphingolipid important for fungal cell division, alkaline tolerance, hyphal formation, and spore germination and, thus, for the regulation of fungal virulence. Present in many fungi, including yeasts, molds, and in dimorphic fungi, fungal glucosylceramide exhibits a characteristic structure synthesized by fungal specific enzymes. Thus, it is envisioned as an important fungal target for compounds with specific and broad-spectrum activity.

## Glucosylceramide: The Sugar-Coated Sphingolipid

**Figure 1:**
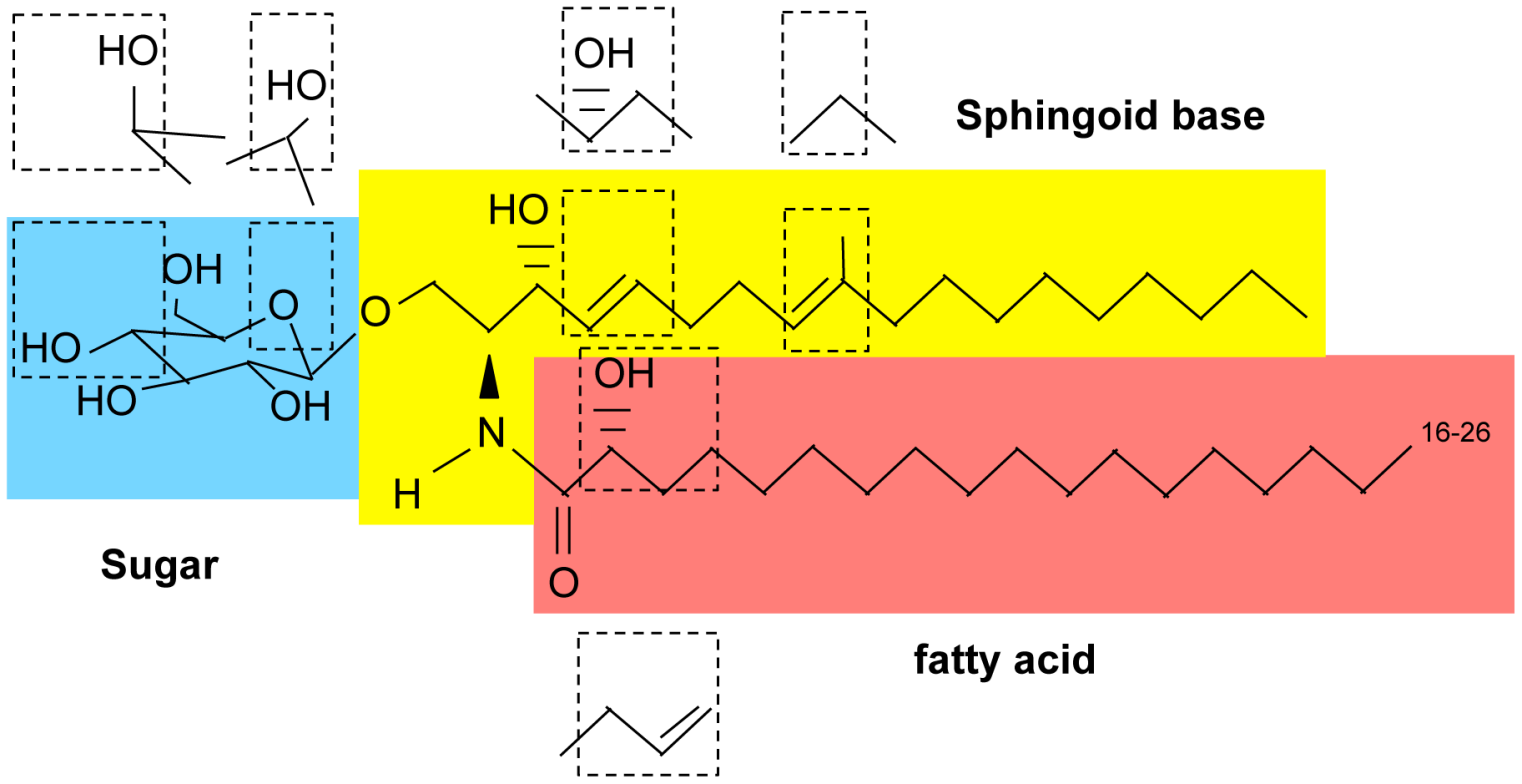
Basic structure of glycosphingolipids. First, a long-chain sphingoid base backbone (distinguished from glycerolipids which have a glycerol backbone) is linked to a fatty acid via an amide bond with the 2-amino group and to a polar head group at the C1 position via an ester bond, forming ceramide. Second, ceramide is linked to a sugar (glucose, galactose, or inositol) via a β-glycosidic bond between the hemiacetal group of the sugar and the C1 hydroxyl group of ceramide.

**Figure 2:**
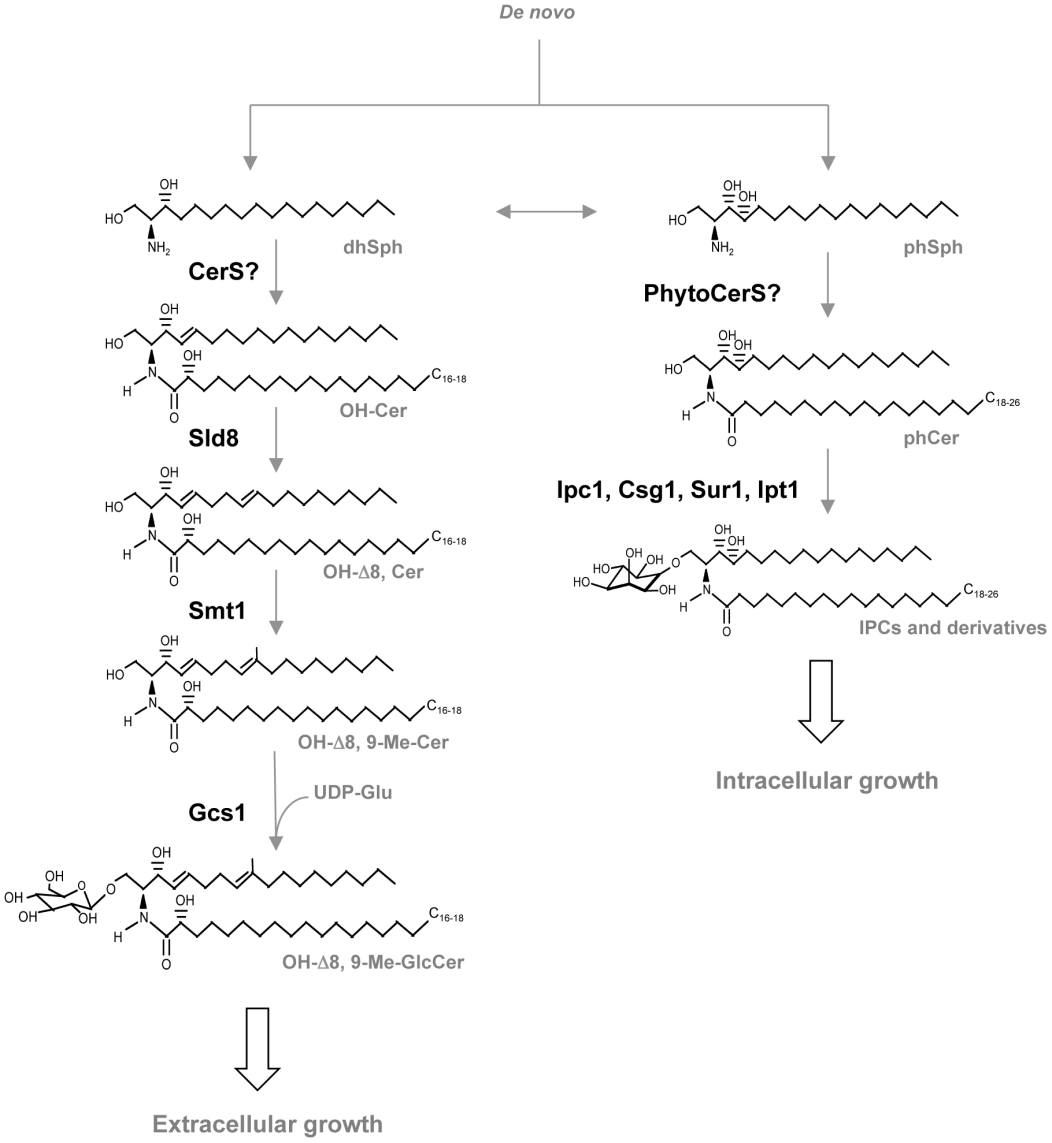
Glycosphingolipid pathway in fungi. dhSph, dihydrosphingosine; CerS, ceramide synthases; OH-Cer, α-hydroxy-ceramide; Sld8, Δ8 desaturase; OH-Δ8-Cer, α-hydroxy-Δ8-ceramide; Smt1, C9-methyl transferase; OH-Δ8, 9-Me-Cer, α-hydroxy-Δ8, 9-methylceramide; Gcs1, glucosylceramide synthase 1; OH-Δ8, 9-Me-GlcCer, α-hydroxy-Δ8, 9-methyl-glucosylceramide; phSph, phytosphingosine; PhytoCerS, phytoceramide synthases; phCer, phytoceramide; Ipc1, inositol-phosphoryl ceramide synthase 1; Csg1, mannosyl phosphorylinositol ceramide synthase regulatory protein; Sur1, mannosyl phosphorylinositol ceramide synthase; Ipt1, inositol phosphotransferase 1; IPC, inositol phosphoryl ceramide.

Glucosylceramide (GlcCer) is a sugar sphingolipid composed of a sphingoid backbone, a fatty acid, and a glucose moiety (Figure 1). Notably, GlcCer is found in plants, fungi, and animals and absent in bacteria and in some eukaryotes such as the baker yeast *Saccharomyces cerevisiae*. In spite of the presence of GlcCer in most organisms, its synthetic pathway and molecular structure varies significantly [1]–[4], resulting in the occurrence of rather unique GlcCer molecular species in different organisms. The final reaction of GlcCer synthesis is catalyzed by the glucosylceramide synthase enzyme (GCS), which transfers a glucose moiety from uridine 5-diphosphate (UDP)-glucose onto the C1 hydroxyl group of ceramide via an oxygen-glycosidic bond (Figure 2).

GlcCer was first isolated from the yeast *Hansenula ciferri* in 1971 [5]. The biochemical characterization of its structure in a variety of fungi, however, became available during the 90s, mainly by the work of two Brazilian groups: one in São Paulo coordinated by Helio Takahashi and one in Rio de Janeiro coordinated by Eliana Barreto-Bergter. During the early 2000s, the German group in Hamburg guided by Ernst Heinz and Dirk Warnecke isolated and characterized the fungal enzymes involved in its synthesis. This made possible genetic and pathobiological studies. The first observation that suggested a critical role for GlcCer in the regulation of virulence of a fungal human pathogen came in 2006, when a *Cryptococcus neoformans* mutant lacking the glucosylceramide synthase (Gcs1) was found to be avirulent in a mouse model [6]. *C. neoformans* is an environmental fungus that upon inhalation into the lung can cause a lethal meningoencephalitis, especially in immunocompromised patients. In the host environment, this fungus is a facultative intracellular pathogen and, as such, it can be found both inside and outside host cells. Once *C. neoformans* enters the host via the respiratory tract, it can survive and replicate in the extracellular environment (e.g., alveolar spaces, bloodstream), or it is engulfed by phagocytic cells (such as macrophages). Once inside phagocytic cells, *C. neoformans* can either survive and replicate in the phagolysome, or it can once again reach the extracellular space by exiting these cells without killing them [7], [8]. Since the extracellular environment is neutral/alkaline whereas the intracellular (phagolysosome) is acidic, adaptation of *C. neoformans* to both environments is a critical feature for its survival and virulence in the host. Intriguingly, different glycosphingolipids (sugar-complexed sphingolipids) have been found to regulate adaptation of *C. neoformans* to both environments [9]. In particular, glycosphingolipids carrying mannose and/or inositol have been found to regulate survival and/or replication of *C. neoformans* in the phagolysosome [10]–[12], whereas synthesis of GlcCer is critical for survival of the fungus in the extracellular environment [6]. Remarkably, loss of the *GCS1* gene in *C. neoformans* produces a fungus (Δ*gcs1*) that is not able to survive in the extracellular environment of the host and, in stark contrast to wild-type clinical isolates of *C. neoformans*, is completely avirulent in an immunocompetent mouse model. Indeed, while clinical isolates of *C. neoformans* (e.g.., wild-type H99 strain) generally cause a lethal meningoencephalitis after dissemination from the lung, mice infected with the Δ*gcs1* (H99-derived strain) are able to mount an effective immune response that ultimately leads to containment of the fungus in a granuloma-like structure. Importantly, these observations unequivocally underscore, first, the critical contribution of the fungal extracellular component to the pathogenicity of *C. neoformans* and, second, the central role played by GlcCer synthesis in regulation of this important aspect of *C. neoformans* virulence.

## Uniqueness of the Chemical Structure of GlcCer

Fungal GlcCer has a very unique chemical structure compared to GlcCers in mammals and plants.

In plants, GlcCer is considered to be the most abundant glycosphingolipid, and its ceramide backbone has the greatest structural variety: seven different sphingoid bases can be linked to each of ten different α-hydroxy fatty acids, yielding to the production of at least 70 GlcCer species. This high variety is not present in fungi or mammals, in which only one or two sphingosine backbones can be attached to few different fatty acids. In fungi, the ceramide backbone in GlcCer is composed of a characteristic Δ8,9 methyl-sphingosine (desaturaded between carbon 8 and 9 and methylated in position 9) mainly linked to 2 hydroxy-octadecanoic acid, occasionally with a trans bond in position 3 (Figure 1 and Figure 2). GlcCer in mammals is almost exclusively made of sphingosine (double bond between carbon 4 and 5 of the sphingoid base) attached to hexadecanoic acid (Figure 1). Moreover, whereas in plants and fungi GlcCer is the end product of the synthetic pathway, in mammals GlcCer is used as a substrate to make more complex glycosphingolipids, such as lactosylceramides and gangliosides. Of note, certain fungi, such as *Aspergillus fumigatus* and others, produce also galactosylceramide, another type of glycosphingolipid in addition to GlcCer in which galactose is attached to ceramide [3] (Figure 1). Interestingly, galactosylceramide is absent in *C. neoformans* [6].

The structure of fungal GlcCer is also unique with respect to other fungal glycospingolipids such as inositol containing sphingolipids. In fact, inositol-containing sphingolipids only carry phytosphingosine as a sphingoid backbone in which carbon 4 is hydroxylated and there is no double bond between carbon 4 and 5 or carbon 8 and 9 and no methyl group on carbon 9 (Figure 1). Interestingly, whereas certain fungi, such as *Candida albicans*, under restrictive conditions can ultimately synthesize inositol-containing sphingolipids from methyl-ceramide, they cannot synthesize GlcCer using phytoceramide [13]. Whether this is common to other fungi is not know. The ceramide backbone of galactosylceramide in fungi is identical to GlcCer although there is a higher proportion of fatty acid with the Δ3-desaturation, but this cannot be generalized because the structure of galactosylceramide has been determined in only a few fungi [14]. On the other hand, it is interesting that its cellular level increases in the yeast phase of dimorphic fungi [14], perhaps suggesting that this glycolipid is also important for fungal growth in the host environment.

All together, these observations highlight the very specific chemical structure of fungal GlcCer compared to GlcCer in other organisms and compared to other fungal glycosphingolipids, and they raise the question of whether this unique chemical structure contributes to the functional significance of GlcCer.

GlcCer being a hydrophobic lipid, it is primarily localized in membranes, and following its synthesis in the Golgi apparatus, fungal GlcCer reaches the plasma membrane. In addition, GlcCer has also been reported in the cell wall [15], [16], especially when cell are dividing. GlcCer is also present intracellularly, and it is a main component of vesicle membranes [17]. These vesicles can be secreted, thus exposing fungal GlcCer to the extracellular environment and to host immune cells. Interestingly, the distribution of fungal GlcCer within cells changes under specific conditions. In fact, it has been shown that during cell division of *C. neoformans*, the level of GlcCer localized in the membranes (plasma membrane and cell wall) increases, particularly at the bud neck [15], perhaps contributing to the structural changes that lead to protrusion and/or fission of the bud. This phenomenon is particularly evident when *C. neoformans* cells are shifted from an acidic to a neutral/alkaline environment.

## Regulation of Alkali Tolerance by GlcCer, Structural Implications, and Relevance to *C. neoformans* Virulence

Recent *in vitro* studies have uncovered a role for GlcCer/Gcs1 in the regulation of alkali tolerance of *C. neoformans*, providing a potential mechanism through which this lipid might affect *C. neoformans* virulence in mice. In fact, it was observed that the Δ*gcs1* strain fails to replicate and grow when the fungal cells are shifted from an acidic to a neutral/alkaline environment. Since the in vitro neutral/alkali condition mimics the host extracellular environment encountered by *C. neoformans* during infection (alveoli and blood stream), it is suggested that it is indeed the inability of the Δ*gcs1* strain to grow in these conditions that determines the failure of this strain to survive in the host extracellular environment, allowing the host to mount an effective immune response and clear the infection [6].

Moreover, the simple methylation of GlcCer seems to be essential for allowing cell division at neutral/alkaline pH, as a *C. neoformans* strain lacking the sphingolipid methyltransferase 1 enzyme (Smt1) responsible for this reaction [18] (Δ*smt1*) is mostly arrested in the stationary phase and cannot proceed through the cell cycle [6], [19]. Mechanistic studies on how methylated-GlcCer regulates cell cycle have been hampered by the fact that this glycosphingolipid is not present in the fungal model *S. cerevisiae*, a yeast in which, contrary to other fungi, cell cycle is very easy to synchronize. The fact that the Δ*gcs1* (GlcCer is absent) and the Δ*smt1* (GlcCer is not methylated) *C. neoformans* strains share the same growth defect and cell cycle arrest phenotypes at neutral/alkaline pH supports the hypothesis that methylation of GlcCer is critical for its function in the regulation of cell cycle progression.

Similarly to the *Δgcs1*, the *Δsmt1* mutant is avirulent in an immunocompetent mouse model [6], strongly suggesting that the loss of alkali tolerance of both Δ*gcs1* and Δ*smt1* is the reason both strains loose pathogenicity in vivo. Of interest, Δ*gcs1* accumulates methylated ceramide which is undetectable in wild-type cells. However, this lipid clearly cannot compensate for the loss of methylated GlcCer in cell cycle progression and cell division, suggesting that both methylation and glycosylation of ceramide is important for cell cycle progression in fungi during alkaline tolerance.

## How Does GlcCer Regulate Alkali Tolerance?

The molecular mechanism by which methylated GlcCer regulates fungal alkali tolerance is still unknown. On the other hand, the fact that in neutral/alkaline conditions GlcCer rapidly concentrates at the plasma membrane, cell wall, and bud neck from the intracellular compartment [20] and that, in its absence, there is no budding [6] suggests that, in these specific extracellular conditions, the presence of GlcCer at the bud neck and in its vicinity becomes important to allow progression through the cell cycle. The presence of methylated GlcCer might be necessary to maintain the proper curvature of the membranes at the bud neck so that the bud can initiate and grow to become the daughter cell. In addition, most of the buds present in the *Δgcs1* and Δ*smt1* cells exposed to alkaline pH are small, suggesting that GlcCer, and particularly methylated GlcCer, may be important for the production of bud membranes.

Also, alkali tolerance is dependent on the activity of membrane proteins that transport solutes, such as iron, hydrogen, bicarbonate, or potassium. The solutes are tightly involved in the regulation and stabilization of the membrane polarization through their movement across the membrane. An integrative model of ion regulation across the membrane [21] and the adaptation of yeast cells to alkaline pH (reviewed in [22]) has been proposed but studied in *S. cerevisiae*. Recent studies in *Can. albicans*, however, suggest that extracellular alkaline pH induces iron starvation and, as a response, the yeast up-regulates a series of transporters and pathways (e.g., Rim 101) to increase iron acquisition [23], [24]. Interestingly, this response to alkaline pH seems to be conserved also in *C. neoformans* [25]., Genomic studies in *C. neoformans* suggest that iron transporters are significantly down-regulated in the absence of methylated GlcCer (Δ*gcs1* and Δ*smt1*) and only when cells are exposed to alkaline and not acidic pH (Singh et al., in preparation). Thus, it is possible that in condition of alkaline stress, methylated GlcCer may be important for iron transporters to function properly.

The regulation of potassium (K+) channels by fungal methylated GlcCer is also an exciting hypothesis because it is well known that K+ are key modulators of transmembrane potential homeostasis and some K+ channels are stimulated by extracellular alkaline pH [26]. Interestingly, one of these K+ channels (TASK-2) is coupled with HCO^−3^ transport in the kidney and the highly hydrophobic region of the transmembrane 4 (TM4) is essential for its alkaline-dependence. An homolog of TASK-2 is present in *C. neoformans*, and it is possible that the methyl group of GlcCer is required to sustain adequate hydrophobicity of the TM4, thus regulating HCO^−3^ transport and transmembrane potential.

It is possible that, under alkaline conditions, the presence of methyl GlcCer in the plasma membrane ensures optimal structural organization/activity of these transporters by affecting membrane potential, organization, stability, fluidity and/or microdomain organization (rafts) of the plasma membrane. Studies in the literature are beginning to address the role and mechanisms by which GlcCer is involved in the organization and functionality of biological membranes.

### Regulation of physical properties of membranes by methyl GlcCer

Glycosphingolipids, including GlcCer, have the propensity to enhance the formation of inverted micelle in the plasma membrane, a phenomenon in which the sugar moieties of the glycosphingolipids of the inner leaflet of membranes undergo self-assembly, forming a micelle with a hydrophilic instead of a hydrophobic core. These inverted micelles alter the characteristic bilayer structure and organization of plasma membranes, allowing a more flexible membrane and a massive influx or efflux of material [27] in and out of the cell during specific cellular insults. Interestingly, this phenomenon is linked to the geometrical and structural features of glycosphingolipids [28], [29] and plants have a variety of possibilities for the regulation of this phenomenon given their large number of GlcCer structures. This phenomenon may be also present in fungi where inverted micelle, by adding physical space in the inner layers of the membrane, may favor the penetration of large molecules, insertion of proteins transporters, or large lipid molecules within the membrane with the resulting effect to favor the growth and elongation of the hyphal (*Can. albicans*) or the growth of the membrane in daughter cells (*C. neoformans*) and/or the germination of spores (dimorphic fungi, molds, *C. neoformans*).

Since methylated GlcCer clearly adds physical space in the hydrophobic pocket of membranes, it is possible that it plays a role in allowing the proper change of orientation/movement of the membranes during cell growth and hyphal elongation, morphogenesis and polarization. As mentioned before, in *C. neoformans* methylated GlcCer is present at the bud neck and it may regulate the formation and the maturation of the bud. Studies in *Can. albicans* and *Fusarium graminearum* have also shown that GlcCer is concentrated at the hyphal tip and that it is important for the mechanical hyphal elongation and morphogenesis. Differently from *C. neoformans*, hyphal elongation is controlled by the C9-methylation of ceramide more than its glycosylation [30], [31]. In contrast, in dimorphic fungi such as *Histoplasma capsulatum*, *Paracoccidioides brasiliensis*, or *Blastomyces dermatiditis*, the production of GlcCer is mostly associated with the yeast-pathogenic form instead of the environmental hyphal form [14], [32]–[34]. The functions of GlcCer in these fungi are not known.

GlcCer has been also implicated in the regulation of spore germination in fungi [35]–[37], but whether the GlcCer pathway regulates these phenomena in *C. neoformans* is not known. However, similarly to its peculiar localization at the bud neck and its role in growth and separation of the daughter cell, GlcCer may also be important for spore growth and separation.

The structural function for GlcCer may be more evident in plants, where the lipid helps the plasma membrane to sustain climate stresses, such as drought and cold, through the synthesis and incorporation of specific molecular species of GlcCer in the plasma membrane, thus increasing hydration and resistance to low temperature. Indeed, different GlcCer molecules are found in plant membrane when exposed to different temperatures or different humidity [38]. Interestingly, importance of GlcCer in protecting against extreme temperatures/humidity may also be relevant to fungi since resistance to drought is particularly important for fungal spores in order to maintain their infectious properties.

### Regulation of membrane microdomains (rafts)

Total membranes isolated from the *C. neoformans* Δ*smt1*, in which GlcCer is present but not methylated, are more leaky, and lipids extracted from these membrane do not form rafts in vitro compared to the lipids extracted from wild-type membranes [19]. In contrast, lipids extracted from membranes isolated from *C. neoformans* Δ*gcs1* strain, in which there is no GlcCer, do form more rafts in vitro than the wild-type membranes, but these rafts are very small in size and they might not be functional in transducing/regulating membrane protein receptors and/or transporters (Del Poeta, unpublished observations). Since the Δ*gcs1* mutant accumulates methylated ceramide, it is possible that this sphingolipid is responsible for assembly of these small lipid rafts through hydrophobic interactions of its methyl group with other membrane-embedded lipids (e.g., ergosterol); on the other hand, it does not seem sufficient for the assembly of larger microdomains. It is possible that the methyl group of GlcCer is necessary but not sufficient to produce a large, complex, lipid raft. A large lipid raft may be produced when the glucose moieties of GlcCer interacts with glucose moieties of other GlcCer molecules and/or with sugar moieties of other sphingolipid molecules (e.g., IPC and its derivatives, such as inositol and mannose). These sugar interactions mediated by intermolecular hydrogen bonds not only form a dense network among the sugar moieties of glycosphingolipids but also will likely contribute to form a compact surface membrane alignment of GlcCer/IPC/MIPC/MIP2C. The observation that 25% of raft lipids in fungus *Histoplasma capsulatum* are made of glycosphingolipids [39] further supports the hypothesis of a sugar network. These forces should also facilitate the entrapment of receptors and other proteins within the lipid rafts. The fact that non-methylated GlcCer (Δ*smt1*) is not capable of promoting raft formation like wild-type suggests that the hydrogen interactions between the sugar moieties are not sufficient, or perhaps they are much weaker if not combined with the hydrophobic interactions mediated by the methyl group of GlcCer. Thus, we propose that the hydrophobic interactions mediated by the methyl group of GlcCer are important to initiate the lipid raft formation by tightening sphingolipids and ergosterol together, whereas the hydrogen interactions between the sugar moieties are important to form large rafts and to entrap other large molecules in it (glycosphingolipids and/or proteins).

## Are Other Aspects of GlcCer Regulation Relevant to Fungal Virulence?

As discussed, GlcCer is present and enriched in the membrane of vesicles that can cross the cell wall, reaching the extracellular space. The presence of these GlcCer-enriched vesicles in the extracellular space might have an important role in pathogenicity of *C. neoformans* and in the modulation of the host response to the fungus. Indeed, these extracellular vesicles are loaded with proteins associated with virulence of *C. neoformans* 40,41 and can impair macrophage functions [42]. Additionally, GlcCer is antigenic, and antibodies against GlcCer have been detected during human cryptococcal infections [15], [43]. Thus, exposure of GlcCer on the surface of the vesicles might also induce a host antibody response. Interestingly, administration of antibodies against GlcCer protects immunocompetent mice from developing a lethal meningitis [44], but their role in protecting immunocompromised hosts, such as HIV-infected patients, is less clear [43]. Even if antibodies against GlcCer would exert a protective role only in condition of immunocompetency, it is proposed that these antibodies may be useful as passive immunization against the infection caused by *Cryptococcus gattii* and certain dimorphic fungi, such as *Histoplasma capsulatum*, *Blastomyces dermatiditis* and *Coccidioides immitis*, which can also afflict immunocompetent individuals.

## Can We Exploit GlcCer for Therapeutics?

All the evidence/observations discussed support a critical role for the synthesis of GlcCer in pathogenicity of *C. neoformans*. Furthermore, since GlcCer may influence virulence of many yeasts, molds, dimorphic fungi, and *Pneumocystis* spp., anti-GlcCer therapy might have a very broad applicability.

One possible therapeutic approach would be to develop small molecule inhibitors that target GlcCer synthesis. In this regard, it is essential to note that key enzymes involved in synthesis of fungal GlcCer are not present in mammalian cells, while the fungal Gcs1 is structurally different from the mammalian homologue (mammalian GCS inhibitors do not affect the activity of the fungal Gcs1 and vice versa). Thus, effort should be expended to identify and test natural or/and synthetic molecules that target these specific fungal enzymes.

Other therapeutic options should consider the use of monoclonal antibodies to fungal GlcCer since they inhibit fungal growth [15], [45] and protect mice against cryptococcal infection [44].

Another therapeutic approach to consider could be to exploit targeting of GlcCer by plants defensins. Defensins are well-known, potent antimicrobial peptides that are able to efficiently kill a variety of microbial cells. Defensins produced by plants and insects interact with fungal GlcCer and kill fungal pathogens in vitro [46]–[49] and in vivo [50].

In conclusion, fungal GlcCer is a virulence determinant with a characteristic chemical structure and synthesized by fungal specific enzymes. Since it is produced by a variety of pathogenic fungi, targeting fungal GlcCer should improve the outcome of fungal infection diseases.
