# Correction: Synthesis and Biological Properties of Fungal Glucosylceramide

**DOI:** 10.1371/journal.ppat.1004886

**Published:** 2015-05-21

**Authors:** Maurizio Del Poeta, Leonardo Nimrichter, Marcio L. Rodrigues, Chiara Luberto

The authors inadvertently omitted a phosphate group from the chemical structure of Inositol phosphorylceramide (IPC) in Fig 2. The authors have provided a corrected version here.

**Fig 2:**
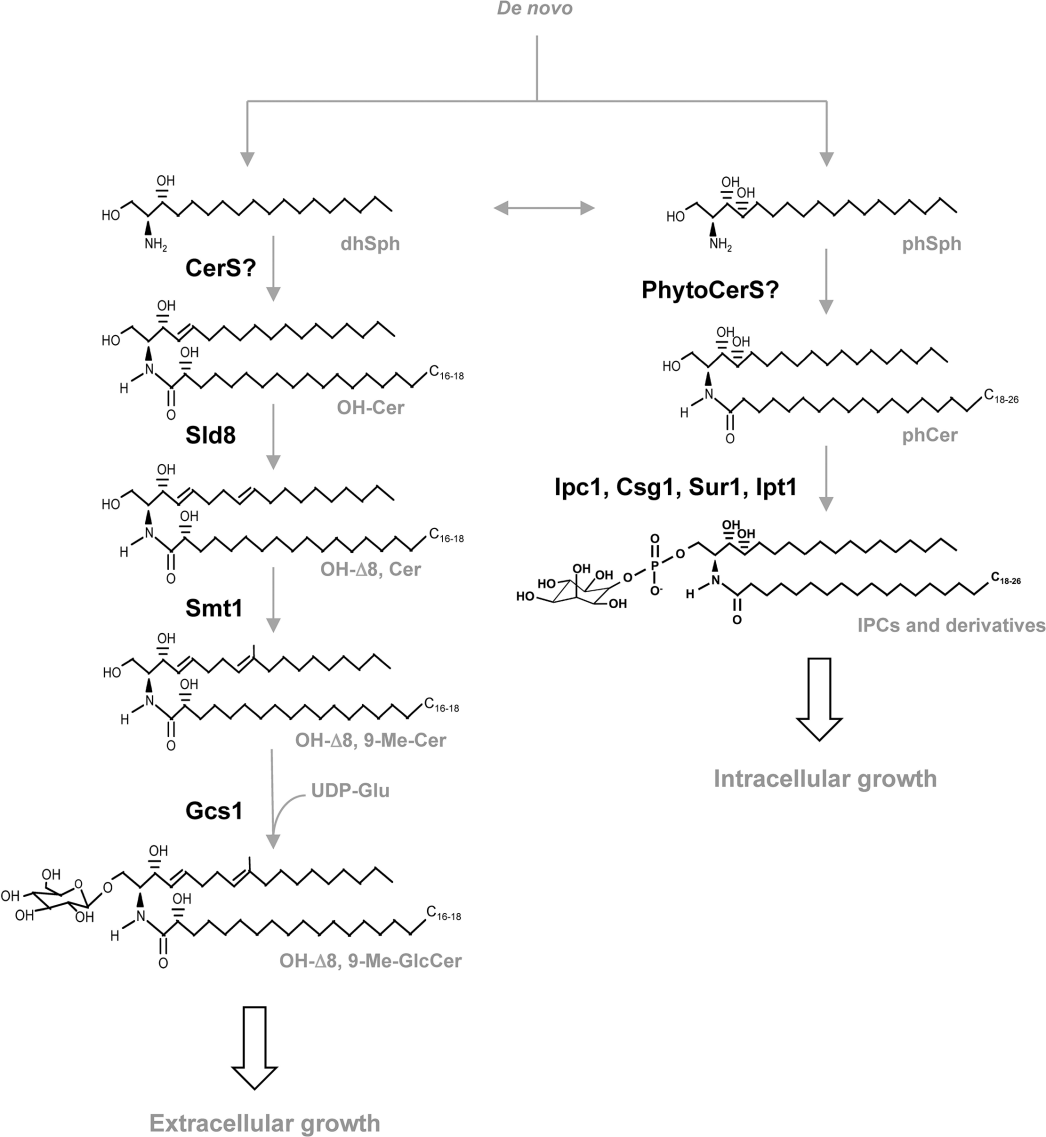
Glycosphingolipid pathway in fungi. dhSph, dihydrosphingosine; CerS, ceramide synthases; OH-Cer, α-hydroxy-ceramide; Sld8, Δ8 desaturase; OH-Δ8-Cer, α-hydroxy-Δ8-ceramide; Smt1, C9-methyl transferase; OH-Δ8, 9-Me-Cer, α-hydroxy-Δ8, 9-methylceramide; Gcs1, glucosylceramide synthase 1; OH-Δ8, 9-Me-GlcCer, α-hydroxy-Δ8, 9-methyl-glucosylceramide; phSph, phytosphingosine; PhytoCerS, phytoceramide synthases; phCer, phytoceramide; Ipc1, inositol-phosphoryl ceramide synthase 1; Csg1, mannosyl phosphorylinositol ceramide synthase regulatory protein; Sur1, mannosyl phosphorylinositol ceramide synthase; Ipt1, inositol phosphotransferase 1; IPC, inositol phosphoryl ceramide.
